# Edge-Computing and Machine-Learning-Based Framework for Software Sensor Development

**DOI:** 10.3390/s22114268

**Published:** 2022-06-03

**Authors:** Pál Péter Hanzelik, Alex Kummer, János Abonyi

**Affiliations:** 1 Enterprise Data Analytics, MOL Group Plc., Október huszonharmadika Street 18, H-1117 Budapest, Hungary; 2 Faculty of Engineering, University of Pannonia, Egyetem Street 10, H-8200 Veszprém, Hungary; kummera@fmt.uni-pannon.hu (A.K.); janos@abonyilab.com (J.A.)

**Keywords:** model maintenance, quality-assurance architecture, Industry 4.0 in Lab, IoT, model lifecycle management

## Abstract

The present research presents a framework that supports the development and operation of machine-learning (ML) algorithms to develop, maintain and manage the whole lifecycle of modeling software sensors related to complex chemical processes. Our motivation is to take advantage of ML and edge computing and offer innovative solutions to the chemical industry for difficult-to-measure laboratory variables. The purpose of software sensor models is to continuously forecast the quality of products to achieve effective quality control, maintain the stable production condition of plants, and support efficient, environmentally friendly, and harmless laboratory work. As a result of the literature review, quite a few ML models have been developed in recent years that support the quality assurance of different types of materials. However, the problems of continuous operation, maintenance and version control of these models have not yet been solved. The method uses ML algorithms and takes advantage of cloud services in an enterprise environment. Industrial 4.0 devices such as the Internet of Things (IoT), edge computing, cloud computing, ML, and artificial intelligence (AI) are core techniques. The article outlines an information system structure and the related methodology based on data from a quality-assurance laboratory. During the development, we encountered several challenges resulting from the continuous development of ML models and the tuning of their parameters. The article discusses the development, version control, validation, lifecycle, and maintenance of ML models and a case study. The developed framework can continuously monitor the performance of the models and increase the amount of data that make up the models. As a result, the most accurate, data-driven and up-to-date models are always available to quality-assurance engineers with this solution.

## 1. Introduction

Software sensors determine critical parameters of complex chemical processes that are difficult to measure. The development and application of software sensors in the chemical industry has been prevalent in the last decade [1]. However, no suitable solution has been developed for their economic operation and lifecycle tracking, so the number of devices is low today. The development of a methodology for cost-, energy- and resource-efficient operation of models facilitates continuous real-time software sensors [2,3]. Several sensors are used in chemical processes to monitor critical process variables such as product quality and process safety. Samples awaiting analysis are taken manually from the process and analyzed in laboratories. Sampling frequencies are often too low for process monitoring and control [4]. The accuracy of models built on databases with relatively small and inadequate standard deviations may give unsatisfactory results. Therefore, the beginning of modeling requires exploration and analysis of basic statistics [5].

Our goal is to present a solution that meets the above criteria and continuously supports the qualification processes. To this end, we have developed a quality-assurance architecture that summarizes the building blocks required to develop such a solution. In addition, we have developed a methodology that supports the application of ML, and we also present a case study detailing the applicability. The technology offers a solution for several different laboratories. In addition to the arguments listed above, the development aims to reduce the environmental impact of laboratory activities and use software sensors in various industrial processes. The various ML algorithms have been developed to calculate critical parameters of the materials based on fast, environmentally friendly, and inexpensive spectroscopic measurements. The ML algorithms can learn essential parts of spectral information that can predict qualitative and quantitative parameters. For example, the chemometrics and ML methods are successful tools for testing the quality and quantity of beers [6]. Furthermore, the combination of Raman spectroscopy and ML is becoming a fast, non-destructive method for verifying the nature or origin of foods [7]. Moreover, another review focuses on biomedical Fourier-transform infrared spectroscopy (FT-IR) applications published between 2009 and 2013, which are used for early detection of cancer by qualitative and quantitative analysis [8]. The excellent results using these algorithms were also obtained when distinguishing the origin of honey [9].

These review articles show how popular the development and application of ML algorithms based on data from laboratory devices are in various industries. First, however, we need to apply state-of-the-art methodologies to ML algorithms, such as Auto ML CRoss Industry Standard Process for Machine Learning (CRISP-ML), which allows these algorithms to be updated. From the literature reviewed, it can be concluded that these models are used many times, but only for a short time, as they deteriorate over time and the development part needs to be restarted. Building and maintaining the right IT industry framework is essential for development and day-to-day application of ML models. Our goal is to develop a framework that can be used in an industrial environment, proposing solutions to the problems outlined above and helping with quality assurance and process control. The developed framework will be developed and tested on oil industry data but can also be used in medicine, the pharmaceutical industry, the food industry and waste management.

To ensure the quality of the products manufactured, samples taken from the production of the company processes must be subjected to quality-assurance laboratory testing. Therefore, a vital issue is predicting the arrival of production samples in the laboratory, which will help allocate resources. The CRoss Industry Standard Process for Data Mining (CRISP-DM) system is used to solve this problem. The system consists of three iteration processes, and an AutoML procedure has been used to allow the comparison and configuration of ML algorithms [10].

The process system engineering (PSE) is now more than 50 years old in the chemical engineering industry, mainly focusing on computer power and the further development of chemical processes using them to promote better plant design, operation, and better product quality for more prosperous, more environmentally friendly, and more efficient production [11]. The key areas such as IoT, cloud-, fog-, edge computing, and ML contribute to a more economical, environmentally friendly, and efficient operation of various processes. ML algorithms have now been adopted to track the quality of multiple industrial processes effectively [12]. In addition to the various ML solutions, increasing the efficiency, development and maintenance of standard data models and ML algorithms is still to be worked out [13]. Due to the complexity of chemical processes, it is challenging to incorporate ML models into continuous or batch production processes. Therefore, improving the integration capacity of corporate governance systems and ML processes is needed. The analysis of processes seems to be a prevalent and innovative solution from the pharmaceutical industry. This topic is called process analytical technology (PAT) [14]. The basis for achieving the primary objectives mentioned above is that the available IoT and edge-computing tools continuously support operational activities with ML models. The models need to be updated based on historical data and practical information. In addition, ML models, such as machines, need maintenance because the models can land or break over time. Therefore, continuous monitoring and maintenance are required for more accurate and robust model results. An industrial data science framework will help address these challenges. Furthermore, companies need to pay more attention to maintaining their ML competencies. In addition to maintenance and supervision, a well-developed architecture and a well-documented framework are key. The edge computing performed by IoT devices communicating with the remote cloud plays an essential role in industrial digitization. The edge-computing architecture can be an ideal solution to minimize delays for intelligent factories and smart cities [15]. The IoT and edge use a gateway to communicate.

A literature review shows that many of the articles use Industry 4.0 devices, but the prevalence of a large number of software sensors is not yet visible. The problem is that an installed software sensor specializes in basic parameters that are difficult to measure. As a result, specialists are required to interpret laboratory measurements. In addition, the maintenance of the model and the tuning of its parameters require continuous monitoring. The purpose of this article is to explore how software sensors can be developed, deployed, and continuously monitored and maintained with edge and cloud computing.

The following main points show the roadmap that will contribute to the methodology we have developed.

- Section 2 describes the related work, overview of cloud- and edge-computing articles used in chemical engineering. The literature review shows that there are quite a few initiatives in these areas, mainly in the healthcare and pharmaceutical industries.
- Section 3 presents the elements of a framework proposed to address the challenges of a general quality-assurance laboratory. The framework helps to develop and maintain models.
- Section 4 presents a case study supporting the work of the quality-assurance laboratory by comparing the performance of different ML models.
- Finally, Section 5 summarizes conclusions and research recommendations.

## 2. Overview of Cloud Computing and Software Sensor Development in Chemical Engineering

This second section presents the importance of the topic, the related literature, and patents. The preferred reporting items for systematic reviews and meta-analyses (PRISMA) methodology is used to review the many scientific sources systematically.

### 2.1. Literature Review

The PRISMA statement includes a report outlining the area of study and assisting the researcher in selecting relevant literature in a systematic review [16]. The analysis makes it easy to review the literature on Scopus or even the Web of Science [17]. Resources related to the topic should be described with a systematic overview and a high degree of methodological detail. The flowchart is an integral part of the methodological description of the PRISMA review. The use of data-driven predictive models is becoming increasingly popular in the engineering and manufacturing sectors. During the literature research, we searched for literature with several word combinations in Scopus. First, the central area of the topic was the edge, computing, software and sensor; the number of articles was 388, of which 14 were chemistry articles. The keywords of other searches were cloud, edge, fog computing and ML or ensemble learning; there were 168 review articles, of which six were chemistry. Next, the chemistry laboratory and ML were chosen as keywords; there were 207 articles, of which 22 were relevant and related to chemical engineering. Finally, there were 17 relevant pieces of literature on edge computing, ML, and chemical engineering. Each combination search shows a few scientific articles on chemical industry software sensors, edge computation, and ML.

**Figure 1:**
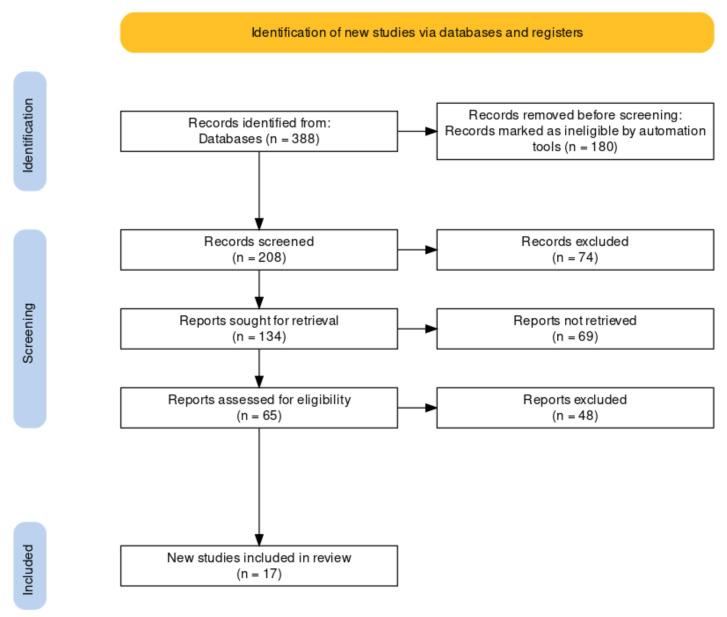
Grouping of articles according to PRISMA methodology. PRISMA chart representing the methodology of the literature review based on the Scopus database. As can be seen, 388 articles started the analysis, but 17 were included in the study.

**Figure 2:**
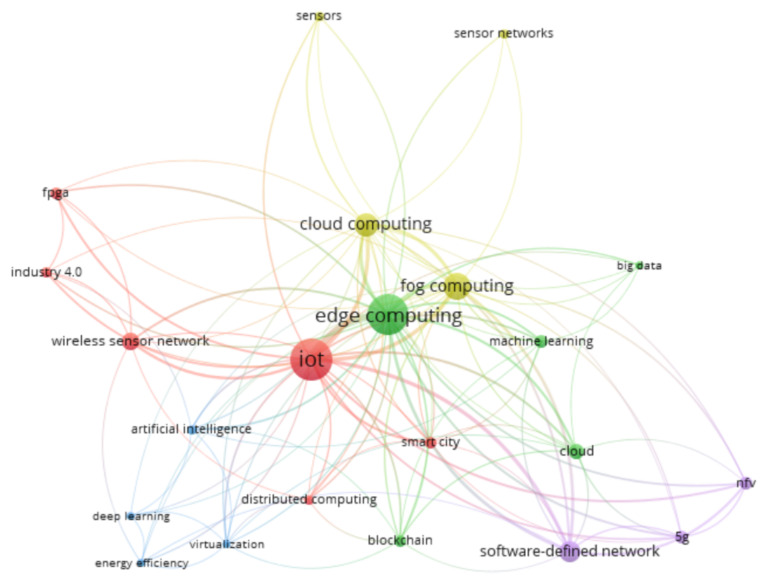
The co-occurrence network of the keywords of “edge computing” and “software sensor”-related articles in the Scopus database. As can be seen, the papers are clustered into four categories. Red shows the IoT, green the edge, yellow fog-, cloud computing and sensors, and blue shows the artificial-intelligent and deep-learning modules.

The selection criteria were the relevant literature on edge computing and software sensors used in the industry. As a result, the articles found on Scopus were processed using the PRISMA methodology (Figure 1). The network diagram summarizes which keywords appear in the scientific journals “edge computing”, “software”, and “sensors” during the first search of 388 articles (Figure 2). Red shows the connection between the network and the application device. The colors green and blue illustrate the devices, methods, and data connecting IoT to applications. The yellow keywords summarize the computing and the sensors connected to extreme computing and IoT.

The five groups shown in the Figure 2 are as follows. The red group contains the wireless sensor networks, and the IoT industrial solutions for the wireless networks. The purple group includes 5G technologies and visualizations. Yellow focuses on the fog- and cloud-computing parts, and the green group deals with ML, edge, and big data. Finally, the blue group is for in-depth learning of artificial intelligence, energy efficiency and visualization. It can also be seen from the network of keywords that the edge computer, IoT and ML algorithms have been intertwined technologies for years. However, little research has been presented on the maintenance and monitoring of the algorithms presented in the literature.

Edge-computing tools play a significant role in the maintenance, onsite access, and developed ML models. The aim of edge computing is to bring cloud resources and services closer to the things that are generating data [18]. Cloud computing provides convenient, on-demand network access to a shared set of configurable computing resources that can be quickly deployed and released with minimal supervision [19]. A group of IoT infrastructures that connect different objects and allow them to be managed, accessed and mined by the data they generate and communicate with other devices [20]. In a broader sense, it extends network connectivity and computing power to objects, devices, sensors, or objects that are not computers [21]. Furthermore, IoT devices play a prominent role in the wireless detection and transmission of signals. Different gateways and devices on the edge of the Internet play a vital role in the operation of modern companies [18].

The digitization of production lines plays a key role in the efficiency of several production units, such as predictive maintenance and quality assurance.

Monitoring the condition and process of data-driven machines in a fog-based framework is of great importance in cyber manufacturing. The communication protocol presented in this article is MTConnect, an open set of standards on which is based standard internet technologies, and Amazon Machine Image (AMI) defines the primary operating system. Manufacturers can use MTConnect to monitor real-time machining and process data, speed, temperature, emergency shutdown, and performance status. Furthermore, because this protocol is implemented as a web service, it is easily accessible to any device that connects to the machine’s network [22]. In addition to fog and cloud calculations, edge calculations are also used in many cases. The point is to carry out onsite operations, make forecasts and thus speed up processes. Recently, prevalent topics such as cloud, edge and fog computing and the IoT are essential for developing smart factories. Osmotic computing has elements that enable more coordinated computing, networking, storage, data transfer, and management between cloud and IoT devices in computing layers of the edge [23].

### 2.2. Related Patents, Trends and Benchmarks

**Figure 3:**
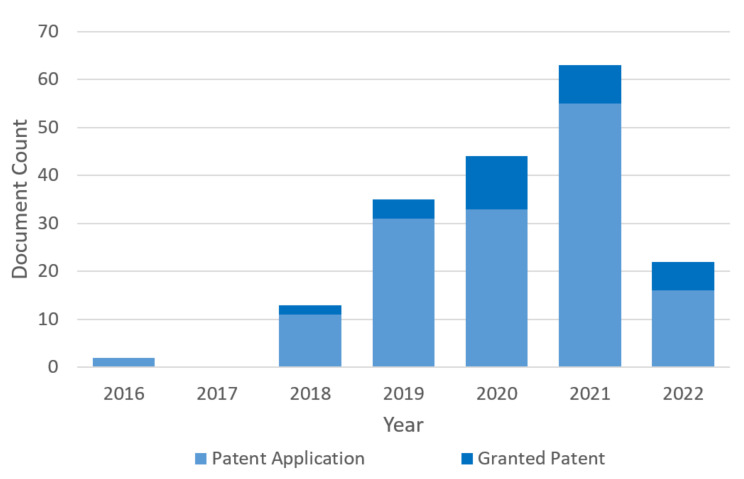
Number of patents in recent years. Patents were searched for on the Lens database; the keywords were “edge computing” and “software sensor”. In addition, there is a significant increase in their annual breakdown, which shows the relevance of the topic today. (Accessed the data at lens.org on 31 March 2022).

It can be seen from the results that this article’s topic is becoming more and more popular year after year, not only from the significant increase in the number of articles in the literature but also the number of patents (Figure 3).

The patents review shows that Fraunhofer Ges Forschung is at the forefront of edge computing and software sensor technology. The Fraunhofer is the world’s leading applied research organization. Prioritizing future-relevant technologies and commercializing its findings in business and industry plays a significant role in the innovation process, such as data innovation development in the different industries, the architecture of the IoT, data-mining and ML algorithms development. This company had 49 patents at the end of 2021, but Hewlett Packard, Version Patent, Sony, Abb, and Intel hold quite a few patents, based on lens.org (accessed on 31 March 2022).

The patents demonstrate the security capabilities of intelligent computing and Industrial IoT devices. For example, one presents a network device that analyzes size and influences packet delivery by a threshold [24]. Furthermore, there are patents in which neural networks transmit the results of each model to the final edge computing. The neural network transformation system can be carried forward using the disguised input data as input to the neural network model. Applying it to the teaching data generated at the first level is the input to the neural level at the next level. The process can be further adapted to pass output data to clients [25]. Another patent discloses disabling live devices that include a processing resource that communicates with a memory resource [26]. There is also a patent that demonstrates the distributed computational mechanism of ML models. The essence of the patent is that it optimizes to run multiple calculations in a hierarchical system, so solving a cost function can give better results [27]. The assignment of ML models to devices is addressed in several patents, one of which presents a method that provides estimates and the score of estimates [28]. Another patent offers a solution for optimizing laboratory procedures. The invention facilitates alternative processes and supports laboratory processes through cost optimization. The essence of the patent is to store data from laboratory processes in an aggregated and structured form that can be easily interpreted and reproduced in laboratories [29].

Based on research in the literature and patents in the field, it can be concluded that ML tools are becoming more widespread in industrial environments. However, there is a tendency in research topics to focus on data collection and model development in the cloud solution, usually using good ML models to ensure quality in minor proof-of-concept (PoC) projects. It can be explained by the fact that maintaining the accuracy of the models requires constant maintenance, as the performance of the models may deteriorate over time. Maintenance is time-consuming and resource-intensive, but this challenge can be solved with the correct methodology, edge- and cloud-computing methods, and appropriate architecture.

## 3. The Proposed Framework

The following section describes the elements of CRISP-ML following principles similar to CRISP-DM and presents the main steps in the sequence of model development (Section 3.1). The concept of cloud-based development of software sensors and its essential tools such as IoT and edge computing are described in Section 3.2. Follow the predictive model markup language (PMML) in Section 3.3 to help you apply, develop, and monitor your models, as well as the lean six sigma principles that are essential for development (Section 3.4).

### 3.1. CRISP-ML for the Sustainability of the Models

**Figure 4:**
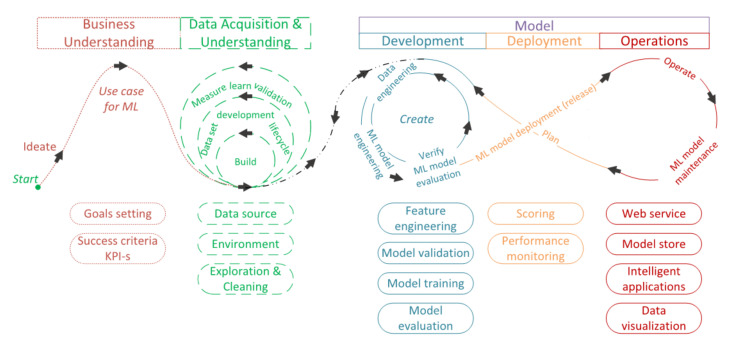
Data science lifecycle management and CRISP-ML. The first part is understanding the business problem (brown) in which goals and success criteria need to be defined. The second stage is understanding the data (green), which involves exploring, cleaning up the data sources and building an environment. The next part is modeling, which has three sub-parts: development of the model (blue), in which it is essential to compete and select different models; deployment of the models (orange), including monitoring the installed models; and operation (purple), which includes data visualization and the development of intelligent applications. The arrows in the figure also illustrate the cyclically of the development.

The following data science technology concept is designed to make data and models available to laboratories and plants at any time of the day. Of course, the goal is to use the latest models as accurately as possible to support chemical processes. The enterprise cloud service needs to be supplemented in a short period with the results of fast, environmentally friendly, and inexpensive measurements of the samples so that predictions can be made from the results obtained quickly for the broad qualification of the products. In addition to uploading data from devices that perform fast measurements, it is also essential to access enterprise resource planning (ERP) data. In addition to data transport, pretreatment, model development, continuous development and maintenance of models are paramount. The application of the CRISP-ML methodology helps in this. The difference between CRISP-DM and CRISP-ML is that the CRISP-DM focuses on data mining and does not cover the application of different ML models inferring in real time over a long period. Furthermore, the CRISP-DM does not give guidance on the quality-assurance methodology. This shortcoming is evident in the standards of information technology and the process models for data mining [13]. The lifecycle of the development of data science models is shown in Figure 4.

To monitor quality assurance in an enterprise environment, it is essential to establish standard process modeling for the development of ML models. In contrast, there are still many developments where this is not happening. Due to the growing demand and recent quality assurance for the models, the CRISP-ML methodology based on the CRISP-DM data-mining model has been developed. CRISP-ML quality-assurance requirements include data quality, model robustness, and expected model performance. The essence of the approach is to articulate risks that could negatively affect application efficiency and the success of ML models. For example, the patterns that make up the models can overwhelm the teaching pattern army, or outlier samples can degrade the accuracy of the models, or incorrectly selected and adjusted models can lead to over-fitting problems. During the prediction of properties that significantly affect the quality of products, the continuous validation of the models is essential, and the application of the CRISP-ML methodology helps in this (Figure 4).

The different colors in Figure 4 show the different parts of the data scientist concept. It is important to note that this figure applies to the development of ML models in general.

- **Business Understanding**
  Projects for the development of ML applications are done by controlling data quality and identifying success criteria. The criteria should be clearly defined and measurable to decide whether the models developed are good or not. In our case, these parameters are the accuracy, reliability, and repeatability of conventional laboratory measurements. In addition to continuous tracking of numbers, it is essential to liaise with the parties designated by the company (e.g., chemical engineers, laboratory development engineers, technicians). For industrial applications, the ML Canvas framework recommends helping define the limitations and application requirements (robustness, scalability). A critical issue in the design of ML models is the quality of the data and the statistical evaluation of the data collected.
- **Data Acquisition and Understanding**
  The development of ML models begins with understanding business processes and issues to be solved. The next phase is followed by a detailed exploration of the datasets and examining the data quality. At the end of the section, it can be determined whether the data research project is feasible or not. If you want a good understanding of the business problem, use an Ishikawa chart that lists the factors that influence the goal and their other influencing factors. At this stage, the success criteria of the models are defined along with measurable key performance indicators (KPIs). Each research topic is determined by a process control or laboratory quality-assurance engineers at each step. ML Canvas supports the forecasting and learning parts of the ML application. In addition, each business site imposes restrictions on model compliance and application boundary conditions. ML Canvas offers the opportunity to outline the solution imagined by ML on a transparent map. The outlined map helps us see what is needed to implement it. In addition, team members provide information to see what else is needed for a successful ML project [30]. Part of the second phase of the CRISP-ML process assumes data sources, data cleaning, and building an environment. In this phase, its main task is to prepare the data for the ML models. The second section also covers service design and data standardization, and appropriate data quality requirements [13]. In the next phase, its main task is to prepare the data for the ML models.
- **Model Development**
  The third phase is the ML model development of CRISP-ML. This is a very iterative process. Occasionally, we may need to review business objectives, define other KPIs, and modify the results of the ML model using available engineering from the available data. In the final phase, the ML workflow is packaged into a process to create repeatable modeling. The modeling phase follows the model evaluation phase, in which the performance of the trained model evaluates on a test dataset. In addition, the robustness of the models should be tested on noisy or poor input data. After testing, a requirement level should be formulated against which ML methods can be applied. In the final phase, before installing the models, the algorithms must meet a success criterion in which ML experts must evaluate the performance [31]. All settings and results for the modeling and evaluation phases should create a detailed document. The introduction of ML models means integrating models into a software system. For example, deploying ML models means that the predictive function is packaged as an interactive dashboard, as a predictive forecast, as a component of the ML model snap-in, into a kernel software architecture, or as a web service endpoint in a distributed system. The implementation of the ML model includes the following tasks: determination of a hardware inference evaluation of the model in a live environment. In addition, one should provide online testing, such as A/B tests, and statistics test, user acceptance and usability testing, and, in extreme cases, plan for model downtime to gradually introduce a new model. Once the ML model is in production, continuous monitoring and maintenance of its performance is essential. A good solution for this is to display the indicators of ML models on a dashboard [31,32], e.g., a depleted model, where the main risk realized is the effect of “model obsolescence”, when the performance of the ML model decreases when it begins to operate on samples of unseen production parameters or data from exceptionally rocky measurements.
- **Model Deployment**
  The next phase is the commissioning of ML models in production. The complexity, size, and complexity of ML models depend on the business problem to be solved [33]. The fourth phase is strongly related to those in front of it, which provides continuous feedback. At this stage, it is essential to select and enter the ML model. One of the main challenges for ML projects is reproducibility and robustness. Therefore, it is crucial to store all metadata related to the data (instrument, measurement setting parameters, environmental conditions, date) and the exact settings of the models (e.g., pre-processing, training, validation dataset division, hyper-parameters, model, structure). All information about the deployed models should be stored using the predictive model markup language (PMML) as well as the machine-learning model operationalization management (MLOps) methodology [34].
- **Model Operations**
  The final modeling phase is the maintenance of installed and continuously running models. In this phase, the available models must be continuously accessed through intelligent applications, and the data must be displayed continuously, e.g., visualization on a dashboard. The use of MLOps is constructive in the third and fourth phases. MLOps is based on hands-on experience designed to monitor the efficient and reliable operation and maintenance in a live environment of the ML models. Cloud infrastructure services provide significant amounts of computing power at a relatively low cost. A significant advantage is that multiple users can share codes and capacities simultaneously. According to the methodology, the models are tested and developed in an isolated experimental system when the model is ready for deployment before being simulated sharply by data scientists and ML engineers to migrate the system. The daily application of ML models is a significant challenge for their application in industrial environments [35]. MLOps and compounds of development and operations (DevOps) are very similar in their efforts to automate and improve production models while meeting standards and requirements. MLOps cover the entire modeling lifecycle, including diagnostics, fine-tuning deployments, and monitoring business metrics [34]. The use of MLOps assists in the installation and automation of ML models, the reproducibility of forecasting, the diagnostics and scalability of models, and the monitoring and, if necessary, management of their interaction. Saved and documented information increases the efficiency, transparency, and explainability of the reproducibility of ML models. One way to do this is to use the “Model Cards Toolkit”. In addition, ML models are increasingly used to perform highly complex tasks. The performance of the models, aided by the version number of the packages used and detailed documentation, helps to understand the task. One way to do this is to create different model cards to help with the structured documentation of the models [36].

The best practice to prevent model performance degradation is to perform the observation task during performance evaluation of the models continuously to determine if retraining is required. Moving models from a monitoring task can lead to updating the ML model. In addition to tracking and retraining, tracking business processes and reflecting on ML models can help determine the mineral composition of oil fields more accurately [37] and make production plants more cost-effective and stable to produce a better product [38].

### 3.2. Concept of Cloud and Edge Based Software Sensor Development

**Figure 5:**
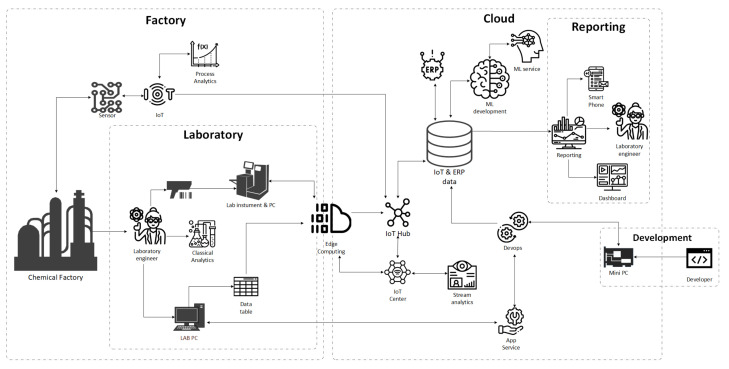
Architecture supporting measurements of the quality control in chemical processes. The dashed lines indicate the boundaries of the two main parts (factory, cloud) and three sub-parts (laboratory, reporting, development), the edge-computing device connects the cloud and onsite area.

The CRISP-ML methodology presented in the previous section requires the development of an appropriate architecture that, in addition to the above, ensures the continuous availability of the models on site and secure and continuous data collection. The external elements of the architecture presented in this section are edge- and cloud-computing solutions. Cloud infrastructure services provide significant amounts of computing power at a relatively low cost. In addition, virtual services are available at a pre-determined hourly rate in these services so that we can pay as much for the service as before. A significant advantage is that multiple users can share codes and capacities at the same time. Cloud computing and MLOps greatly facilitate the development, monitoring, and subsequent operation of ML models. Our concept is essential for storing laboratory data in the cloud and for the joint handling of data related to the manufacturing process, such as temperature, pressure, and analytical measurements. Data are transferred from laboratories using various edge-computing devices and from production using IoT. The data analysis thus collected can provide rapid support in product quality using the results of ML algorithms and the condition of the machines involved in production. Furthermore, data transmission and models should work seamlessly in terms of data availability. The architecture related to the concept is illustrated in Figure 5. The figure shows that the relevant architecture consists of two main parts (factory, cloud) and three sub-parts (laboratory, reporting, development). The main parts of the environment are defined by the factory process tracking and intervention, by the laboratory data collection and model running on-prem environment, while building the data pipeline, algorithms development, ML services, model monitoring and reporting are conducted online.

- **Process tracking and intervention**
  Process control colleagues constantly monitor industrial sensors with various software that connects to IoT devices via a LAN cable. Process engineers monitor various parameters such as temperature, pressure, and material flow rate. From these parameters, the best conclusions can be drawn about the products’ goodness. They can also get accurate results by predicting ML models of laboratory equipment. The samples of the process are transported to the laboratory, where colleagues prepare the samples and perform measurements using classical or rapid innovative measurement techniques.
- **Data collection and model running on the edge**
  The results of the classical measurements are manually uploaded to the enterprise system. Data entry for rapid measurements is completed with a QR code reader for easier, faster and simpler use. The computing devices in the field are connected to the edge device with a LAN cable, which transmits the data to the cloud. On lab computers, colleagues can run ML models developed in the cloud and tested on a minicomputer. As the figure shows, the critical part of the architecture is the edge computer. This device establishes a connection between the factory and the cloud service to be real-time and continuous data transfer.
- **Machine-learning model building and development in cloud**
  Another critical part of the architecture is the IoT and ERP data market, where data engineers carefully compile data from different sources, which data researchers will then process. ML models are being developed in a cloud environment, moving into cutting-edge computing through data flow analysis and the IoT center. Maintenance of models and continuous monitoring of their performance is critical. It is essential for the production unit in the field always to have the best models available. Maintenance of models and constant monitoring of their performance is vital. It is necessary for laboratories always to have the best models available. By validating laboratory measurements and ML models, robust and efficient models can be developed that must be monitored continuously and intervened when warranted. Testing new, better models before the live operation for continuous model development is essential. It is imperative to separate these tests from the existing system completely.
- **Machine-learning model testing**
  The new models are tested through a virtual unit, simulated as if sharp samples were running. In all cases, experts in data science and the business process should perform this activity with due care. Then, when the models have proven to be suitable, they can deploy the new ones on the edge device with an update. The great strength of the architecture is the continuous development and application of ML models, which we can teach and update every minute.
- **Reporting and quality control**
  Applications in Industry 4.0 solutions allow continuous evaluation and the real-time monitoring of results. Reporting professionals can easily track the results of a plethora of lab samples on a dashboard, even on a smartphone. In addition, the dashboards are easy to customize and provide users with live data at any time.

**Figure 6:**
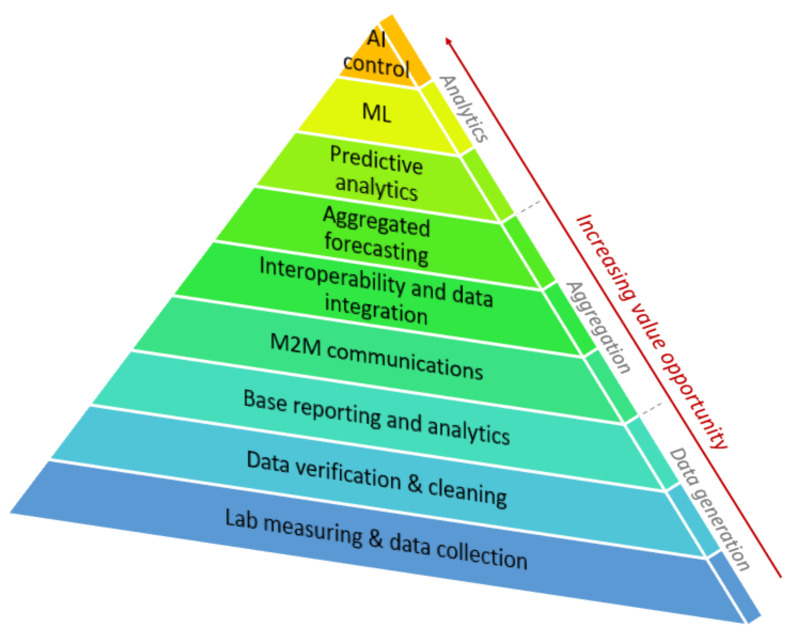
Level of the data processing. The layers show the integration of data into the corporate control system. The higher the level of the pyramid, the more complex the data-based processes.

Continuous data collection aims to make the most efficient use of data from industrial units to monitor processes. For example, the intermediate component of the oil fields or the different element content of the product is essential. In Figure 6, the layers show the different levels of data processing. The first level is the secure collection and transmission of data. After collecting the laboratory data, the second level is to clean the data and prepare the fundamental analyses and reports. The fourth level is aggregation, which begins with communication between machines and then includes data integration and aggregation forecasting. Finally, the level of analysis begins with predictive analysis, then with ML, and finally with AI. The data from the IoT or edge device units are sent as a pyramid, and the point is that the measured raw data are under AI control.

### 3.3. Secure Data Collection and Running on the Edge Device

An essential aspect of the development project is to make the developed models available for production and certification even if something goes wrong between the cloud and the terrain. If we have some issue with edge-computing, troubleshooting is also easier. Edge computational analysis and knowledge generation occurs at or near the source of data and computational performance, away from centralized points toward the edges of the network. Edge computing should emphasize that this model does not rely on data centers, but has ready-packed models developed in the cloud. Edge computing is a distributed computing platform that brings computing and data storage closer to shortening response times and minimizing potential distance challenges and problems. As a result, it increases the speed and efficiency of responding to information. This computing platform is similar to a cloud-based platform, only closer to applications. Edge computing analyzes some data from IoT devices on the edge of the local network and transfers them to the cloud. In the technique we have developed, laboratory information management system (LIMS) and ERP data must be available on the edge device in addition to the measurement results. Therefore, selecting the optimal edge device in the market is crucial. Many manufacturers produce a variety of sharps, the parameters of which can vary significantly. The edge device of our choice is a mini personal computer (PC). An essential aspect of the research was that the device could be used in extreme field conditions (the temperature varies between −40 °C and 85 °C), not just in the laboratory. The carefully selected edge tool securely transmits the collected data to the cloud and stores and runs the models packaged after the appropriate command.

A possible solution to eliminate possible attacks is to use block-chain technology. The technology offers a suitable capability for secure data transfer and ML model deployment to IoT and edge devices [39]. However, there are other secure solutions besides or with block-chains.

### 3.4. Implementation of Software Sensor and Machine-Learning Model Monitoring

Once the models are developed, their maintenance is critical because they can become obsolete over time, and their performance decreases compared to their development. Therefore, to always have a suitable model available in the field, we monitor the accuracy of the models and the measured performance (Figure 4, *Deployment, Operations*).

**Figure 7:**
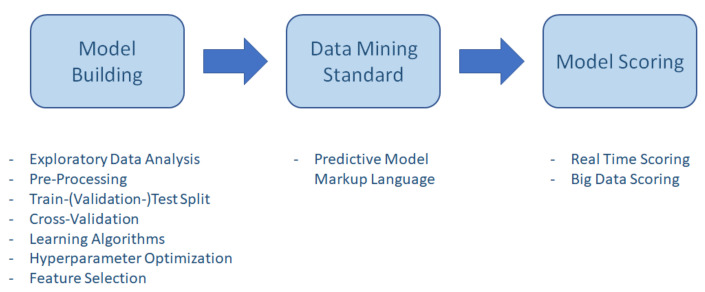
Predictive model markup language-based data-mining activity. The three main sections show the main stages in the development of the models.

PMML is an XML-based specification for the representation of statistical and data-mining models [40]. This can be used in the CRISP-ML approach that makes appropriate ML models available for quality assurance, helping the development, deployment and operation of ML models (Figure 4, *Development, Deployment, Operations*) [41]. ML model version numbers, settings, data dictionary and conversion, developer information, licenses, and package release numbers are all built in. PMML is an accessible markup language created for ML models. PMML is similar to HTML, but it is the hypertext markup language for web pages. PMML is an XML derivative developed specifically by the developers of the Data-Mining Group (DMG) consortium to provide statistical and data mining for sharing between software and programs [40]. The great advantage of PMML is that it is vendor-neutral and conforms to any standard that is widely accepted and easy to use as a markup language for enterprise databases [42]. This reduces the potential for conflict and an open-ended platform that allows ML models to be developed and deployed. PMML is an open access de facto standard for storing and exchanging predictive models [43], such as cluster models, regression models, trees, or supporting vector machines. In addition, development and deployment are separate, allowing data scientists and software professionals to develop models separately and quickly (Figure 7). With the power of a markup language, you can decide in minutes whether or not a model can be put into service for years. With PMML, models can be easily logged and consist of the following main components: header, data dictionary, data transformations, and model. Of course, the pre-processing and post-model post-processing steps can also be stored before the models, and the model explanation allows performance to be evaluated. The PMML represents not only a wide range of statistical techniques, but also the data transformations needed to turn input data and raw data into meaningful functions [44].

The performance of the models can be measured by various tools such as lean six sigma (LSS) and statistical process control (SPC) [45]. Improving the efficiency of processes is essential for environmental and economic reasons. The increase in efficiency is due to the combined effect of the LSS principle, and the ML algorithms [46]. Six sigma can be used to measure product quality and ML model performance. Since the accuracy of a ML algorithm can be quantified, the goal is continuous improvement. The goal of the models is to reach the accuracy of six sigma, so we can reduce mistake product volume, which will increase revenue. It is essential to mention that all these findings also play an essential role in developing the models. The continuous data collection, model re-learning, and algorithm experiences contribute to achieving the best predictive results.

These three metrics are key indicators of each laboratory measurement where the standards are provided. These numbers also affect the goodness of the models, as the reference data pertain to these numbers [45]. An essential tool in enterprise quality management is SPC [47]. It can effectively and verifiably distinguish abnormal fluctuations in product quality. Therefore, intelligent and efficient SPC is of great importance to factories, especially Industry 4.0 [48]. The key property of SPC is that it focuses on histogram pattern recognition and can mathematically support the detection of manufacturing differences [48]. Different pipelines can be used to easily track the performance of the SPC models [49]. The continuous integration/continuous delivery (CI/CD) process introduces monitoring and automation to improve the application development process, especially during the integration and testing phase, and then further during shipping and installation. The CI/CD is a methodology in software development that combines continuous integration with continuous delivery. The added value of CI/CD pipelines is achieved through automation, but it is even possible to perform each CI/CD process step manually [50]. The CI/CD automation keeps the deployed ML models up to date without causing disruptions to production (Figure 4, *Deployment*) [51].

The main elements of the proposed framework are: following the CRISP-ML methodology, and applying it to the developed and validated ML models using MLOps, PMML for model tracking and archiving, CI/CD pipeline for easier use of the models. One should select the appropriate cloud service and edge device for the required devices, considering computing needs and connectivity options, and choose the right reporting tool if it has the option of even a smartphone-compatible dashboard service.

## 4. Case Study

This section presents a study that provides an opportunity for complex companies to predict difficult-to-measure and critical parameters. During the development, the possible deterioration of the quality of the models should be monitored, in which the CRISP-ML approach can help. This section describes the reason for the development (Section 4.1), the technology and the tasks encountered (Section 4.2), method implementation (Section 4.3), the ML models used (Section 4.4) and lessons learned (Section 4.5) by this case study.

### 4.1. Background

In addition to the production of motor fuels, the production plants of integrated oil companies also produce lubricating greases. Therefore, the product range of the bread material production unit is very diverse. Sourcing requirements and standards determine the exact product mix. In the case of ML algorithms, it is essential to emphasize that the number of models is determined by the number of products and their parameters. Therefore, the development and maintenance of ML models is essential for companies. The best version of the models should always be available on site. The wide range of products poses a severe challenge to the continuous presence of the best models. Without CRISP-ML, MLOps and PMML there would be plenty of untraceable models that could not be operated in the long run. The company has a data team responsible for moving data, developing models, maintaining and reporting. Measuring the penetration and metal content parameters of lubricants and greases under operating conditions has so far proved impossible. However, ML models built on laboratory measurements have proven that this can be done with software sensors installed in the right place in plants. Onsite deployment of live computing tools and cloud computing is essential for developing quality-assurance models.

### 4.2. Technology Task

The development goal is to create a unique application that can automate the work in the laboratory and help the day-to-day activity of the laboratory colleagues. Furthermore, another goal is to verify and collect laboratory data and production data of the process. The continuous monitoring of difficult-to-measure parameters with software sensor lines provides our plants with accurate material flow quality information or well analysis of drilling samples. Furthermore, on the well samples, can we use for this methodology prediction for the mineral composition.

Reducing the response time of laboratories and measurements using less hazardous substances is of paramount importance in laboratory developments. Our goal is to obtain the most information out of a lab sample and do it all in the fastest way possible. Fast and non-destructive measurements include various spectroscopic measurements such as infrared (IR), Raman spectroscopy, X-ray, and gas chromatography. The essence of these measurements is that the device makes a curve from a small amount of material, which has much more helpful information about the samples. Furthermore, the measurements do not require the use of hazardous substances. The measurement process can be automated. If the appropriate sample is prepared, then devices can be left alone until all the completed measurements have been completed. The measurements listed above provide different information about chemicals, so storing these measurements in a standard “data lake” is an essential part of laboratory development. The Industry 4.0 devices help to store measurement results in one place. For example, the edge computing or IoT sensors described above are essential for moving data. Laboratory measurements can easily connect to the corporate data, even with minute updates.

### 4.3. Framework Implementation

An essential aspect in the construction of models is the quality of the parameter upon which the model can be built. In addition, an important consideration is where and how a given parameter can predict. Therefore, the models for laboratory measurements help the installation of software sensors for operational and even drilling intelligent sensors. The first phase of the CRISP-ML methodology Figure 4, *Business Understanding* business task, is to understand that the estimation of nitrogen from the operating parameters and the quartz content from the drilling rock samples gives great potential for estimating ML models. The success criterion of nitrogen model estimation was determined by the reproducibility value of the classical measurement in the quartz model, although the degree of error of the model and the speed was associated with the estimation. The developed model meets the first phase of the CRISP-ML criteria in both cases. In the second phase, in understanding the data, an important test was whether, in both cases, the traditional measurement could be replaced by a fast, non-destructive model, and the models built in this way would be a good starting point for the installation of later software sensors. The data understanding phase (Figure 4, *Data Understanding*), regards measurement data and what errors we have in our measurements (reproducibility, repeatability). Data sources in both cases were the edge tool and ERP and LIMS, respectively. During the modeling, we used particular train–test splitting for both target variables, which can monitor the data distribution from the two datasets. The distribution of the train and test datasets with the application was similar. We used 10-fold cross-validation (10-cv) to develop the models, and PMML to deploy the models. Colleagues can track the results and accuracy of deployed models using a visualization tool, PC application, web browser, or even a smartphone.

**Figure 8:**
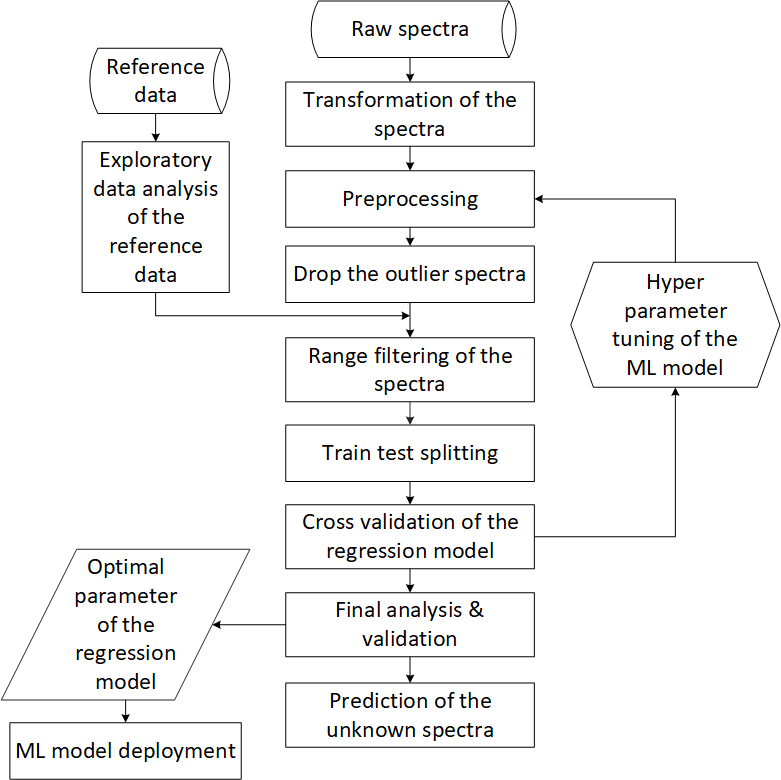
Major stages in the development of ML models. The parts are considered general laboratory, quality assurance and industry independence.

Newer and newer measurements from the edge device must be reviewed through validation (Figure 5, *Edge computing*). ML maintenance shows whether the sample is worth incorporating into the model or not. In addition, newer and newer samples help track the performance of models currently in service (Figure 5, *Reporting*). The built models must be able to handle such changes, so the models are maintained, and the data are displayed through an application (Figure 5, *Reporting*). The model development steps for a parameter of material flow are shown in the figure below (Figure 8).

**Figure 9:**
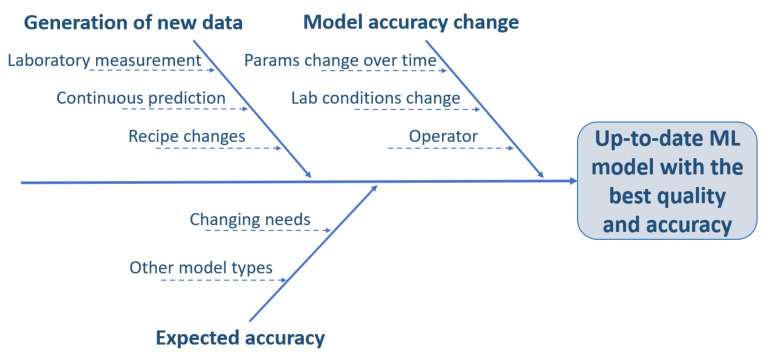
Ishikawa diagram related to the development of ML models. The accuracy and applicability of the ML model can be influenced by the three main factors, which are affected by two or three things.

The main parts of the development of ML models outlining the simplified steps of data processing and modeling are exploratory data analysis (EDA), pre-processing, outlier detection, train–test splitting, with a special technique that considers the distribution of the target variable. Then, the iteration process shows the fine-tuning of the model parameters, and finally, low-error models with the appropriate settings are deployed. This process must be set separately for each parameter (nitrogen, quartz content etc.) in each family of laboratory samples. Laboratory results from measurements can often not be used directly for interpretation or modeling. It must be tied to some calibration to understand business, or in many cases, some mathematical technique must be used (Figure 4, *Business Understanding*), in all cases involving the business colleagues. To determine what influences specific parameters the most, we use the Ishikawa diagram mentioned in the previous Section 3.1) (Figure 4, *Data Acquisition*), which shows the target variable and the factors and sub-factors that most influence it. Following the CRISP-ML methodology, this figure is constantly expanding. Therefore, the role of each factor in the design of the models should be examined. If the accuracy of the model can be easily affected by these factors, the model must be prepared to solve these challenges with robustness (Figure 9).

**Figure 10:**
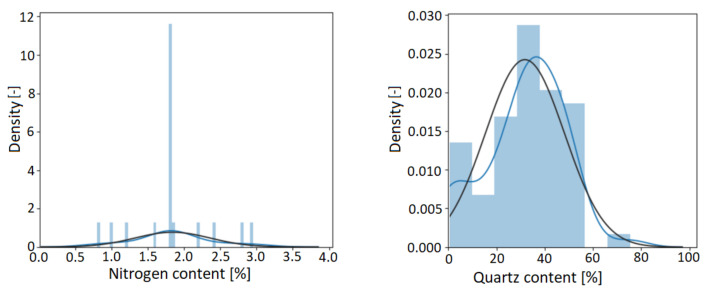
Histogram of the target variables. The left diagram shows the nitrogen content related to lubricants, and the right diagram represents the quartz content of the Exploration and Production laboratory. The black curves show the normal distribution of the given modeling dataset, and the blue is the actual distribution.

The distribution of the modeling datasets of the ML models constructed in the two laboratories presented in the case study is illustrated in Figure 10. The *x*-axis of the figure shows the given property to be measured as a percentage, and the *y*-axis shows the density. The quartz content in the upstream laboratory and the nitrogen content in the lubricant laboratories are measured. The distribution of quartz data is much more favorable for modeling than the nitrogen content. It can be explained by the fact that the variability of the nitrogen content during stable operations is much smaller than the quartz content of the rock sample from several oil fields Figure 4, *Business and Data Understanding*. Tuning the models and testing their robustness for variables with a high skewness (>3) value is paramount. In addition to calibration samples, other samples should be included in the model, such as products manufactured under extreme manufacturing conditions or products of poor quality produced under laboratory conditions.

The quartz content is based on the X-ray diffraction measurement, and the nitrogen content is the target variable from the Kjeldahl measurement method. In both cases, the FT-IR spectra give the predictor dataset of the model. The ML models are validated with 10-fold cross-validation.

### 4.4. Evaluation and Type of ML Models

During the real-time operation of ML models, it is essential to continuously check the accuracy of the models to determine when a particular model is considered excellent, good, or unsuitable. When evaluating the models, the three-“R” index of the classical measurements must be considered [52]. In general, a model is considered adequate if the prediction accuracy of the new samples is within the reproducibility limit. Models that exceed the reproducibility value of conventional measurements are considered unsuitable (Figure 4, *Business Understanding*). Monitoring models allows them to be ranked based on percentage overshoot. The monitoring system displays the models with the most significant errors at the beginning of the ranking, in which case the intervention is urgent (Figure 4, *Deployment of the Model*). Model KPIs are similar to different metrics in traditional laboratory measurement techniques. For decades, we have used circular measurements of various standards to validate devices periodically. Therefore, the calculations are very similar when using the indicators of the ML models. Correlation coefficient ($R^{2}$), root-mean-square deviation (RMSE), and relative percent differences (RPD) are important indicators for tracking ML models. In this study, we compare three different algorithms to estimate the given parameter with the best algorithm. A special linear regression is complemented by a particular calculation that can also handle non-linearity problems. The other two tree-based algorithms are prevalent random forest and extreme gradient boosting. A vital consideration in the selection was to choose an algorithm that would qualify the samples. During the measurements, the ML algorithms must be robust, not sensitive to outlier samples (Figure 8, *EDA*), and the methodology of the competing algorithms is different. The three algorithms must be optimized and tested for each target variable, and then the best of the three is implemented on the edge tool. For installed models, the model type may have changed during development.

A brief theoretical overview of the three model types selected is provided below. The partial least squares regression (PLSR) model is possible for allowing the score matrix to represent the data matrix. A simplified model would consist of a regression between the scores for the X and Y block [53].

(1) $$X=S_{X}L_{X}^{\prime }+E_{X}$$

One can build the outer relation for the Y block in the same way:

(2) $$Y=S_{Y}L_{Y}^{\prime }+E_{Y}\;,$$

where S is the score, L is loading matrix and E represents errors.

Partial least squares has been gaining popularity as a multivariate data analysis tool due to its ability to cater for noisy, co-linear and incomplete datasets. PLSR was supplemented by a nonlinear iterative partial least squares (NIPALS) algorithm supplemented by a nonlinear iterative calculation, based on a recursive computation of co-variance matrices and gradient-based techniques to compute eigenvectors of the relevant matrices [54].

Random Forest is a tree-based algorithm that combines the outputs of multiple decision trees to create the final output. The term “random” is because this algorithm is a forest of randomly generated decision trees. The simpler decision tree algorithm was not chosen because it has a significant drawback that causes over-matching, which can be limited in implementing random forest regression (RFR). Another significant advantage is that the Random Forest algorithm can be very fast and robust compared to other algorithms.

The following formula shows how to calculate the RFR:

(3) $$F\left(x_{t}\right)=\frac{1}{B}\sum_{i=0}^{B}F_{i}\left(x_{t}\right)$$

where:

- $x_{t}$ = test samples
- *B* = Time for random sampling with replacement from the original data. This sample functions as the training set for growing the tree.
- $F_{i}$ = a function of each decision tree, each tree being grown as much as possible without pruning.
- *F* = Outputs function; in the case of a regression problem, we take the average of the predictions for each tree.

Extreme gradient boosting (XGBoost) is a popular algorithm for gradient-increased trees. The method of the algorithm tries to accurately predict the desired target variable by combining estimates from simpler, weaker models. XGBoost minimizes the regularized (L1 and L2) objective function, which combines a convex loss function (the difference between predicted and target outputs) and a penalty term for the complexity of the model. The training is completed iteratively by adding new trees, which predicts the remnants or defects of the previous trees, which are then combined with the previous trees to make the final forecast. In addition to using a unique method to build and prune trees, it also has custom optimization. It is an excellent advantage as it makes computing faster on substantial datasets.

(4) $$S=\frac{\sum_{i=1}^{n}R_{i}^{2}}{\sum_{i=1}^{n}\left[PP_{i}\left(1-PP_{i}\right)\right]+\lambda }$$

where:

- S = Similarity Score
- $R_{i}$ = Residual, which is the difference between actual value and predicted value (observed value − predicted value)
- PP = Previous probability is the probability of an event calculated at a previous step. The initial probability is assumed to be 0.5 for every observation, which is used to build the first tree. For any subsequent trees, the previous probability is recalculated based on initial prediction and predictions from all prior trees.
- λ = Lambda is a regularization parameter. Increasing it reduces the effect on the leaves with little observation, while many observations have little effect on the leaves.

An essential element in the development of robust models is the examination of the sensitivity of the models. Sensitivity analyses evaluate changes in system inputs and the individual effects of each variable on the output and provide information about the different impacts of each variable tested. In addition, it is essential to produce a sufficient number of samples and rare samples to install good models. Extreme samples can be prepared by the design of the experiment (DoE) for the latter process; these samples help to achieve the robustness of the models. During development, we calculated the accuracy of the models for each laboratory property for validation and test datasets. The models were optimized so that KPIs did not differ significantly in training, validation and test datasets, thus protecting the models from over-fitting.

The following two tables summarize the accuracy of the ML models built on the two tested properties. It is important to note that the pretreatment of the spectra before the three model types was the same for both properties (Table 1 and Table 2). The ‘10-cv’ ten-fold cross-validation results are represented by the ‘perf.’ metric that represents the performance of the model on samples not used in the teaching of the models. From the results presented in these two tables, it can be concluded that XGBoost is overfitted and performs the worst despite hyper-parameter tuning. PLSR shows a balanced average performance, and the RFR is the best-tuned ML model out of the three models. These model results show that we can discuss the two important parameters included in the study with ML models. By applying the models, we can determine specific key parameters much faster, with which we are already able to reduce the load and response time of the laboratory significantly. Furthermore, after testing the developed models, the installation of factory software sensors can be solved with the involvement of factory technologists. In the case of lubricants, the development provides support for where to install sensors, while in the case of upstream wells, software sensors can be allowed in the wells. The parameters required by the plant are designed to reduce overall equipment effectiveness (OEE) during lubricant production and to find the proper reservoir for upstream drilling. With the help of the models, scrap products are reduced during the production of lubricants, and in the case of quartz models, we obtain a more accurate picture of the geological formations.

The models are currently available to laboratories monitored through reporting and web application. For the samples examined, there are different ranges at which the system indicates the difference between the prediction and the classical measurement. After ten indications, the web application automatically indicates the validation required for the ML model. Then, our data scientist colleagues review the poorly predicted samples and develop the model if they deem fit.

### 4.5. Lessons Learned

The advanced analytical models of the production and research laboratories can quickly measure many more samples. The architecture presented above and the models developed can reduce laboratory workload and facilitate measurements with lower health and safety executive (HSE) risk. Instead of classical measurements containing difficult-to-measure, hazardous materials, the accuracy of ML algorithms deployed on edge-computing devices for different qualification properties can change significantly over time. This solution may cause changes in the production program, such as different raw materials or new geological rock samples not yet known by the model. The accuracy of the models may also be affected by the operating time of the devices, the degradation of the light sources, the relocation of the devices within the laboratory, or the extreme measurement conditions of the measurement of the samples (e.g., human factor, temperature, humidity). Fortunately, the infrared measurement technique presented in the present study is less sensitive to measurement conditions and instrument ageing. However, changes in sample quality can easily affect the accuracy of models. Checking the accuracy of models should become a daily practice for manufacturing and research laboratory engineers. They can report to data scientists or model developers who can solve the problem quickly. After installing the system, monitoring and maintaining the models of the edge device is also essential. In addition, the tool is responsible for real-time data transfer and accessing the latest models onsite. The edge device selected in the study is the MOXA-8200, the configuration and operation of which posed a severe challenge during development. MOXA is an excellent tool for collecting data and managing a few models, but increasing the number of models results in severe limitations when using the device. The market for edge-computing devices is changing very dynamically, so it is worth reviewing the devices used from time to time. The tool tested in the case study was hired from a local support company, so it is easy to ensure that the best tool is always onsite.

The case study presented in this section can estimate difficult-to-measure, problematic parameters using different ML algorithms. The strength of the models developed is that the right ones are constantly available. Tracking and keeping models up to date is a challenge for research and manufacturing laboratories, with cloud and edge-computing techniques providing a solution. They offer turnkey solutions for data transfer, design, model development and deployment. However, the two techniques present a severe opportunity and difficulty for the safe and continuous supply of industrial processes. Therefore, it is essential to ensure the real-time accuracy and availability of the models (Figure 5).

Applying the CRISP-ML methodology presented in this article significantly reduces the time required to collect, create, and develop data and deploy ML models. Experience has shown that the steps of the first models took a total of 150 working hours by three colleagues, a laboratory technician, a data scientist, and a technologist. Furthermore, introducing the first ML model took about 60 working hours from a data scientist and data engineer. Building a new average ML model from the beginning with CRISP-ML involves data mining, cleaning, outlier filtering, and creating a basic model of about two and a half hours. Testing and commissioning takes one and a half hours. Finally, it takes another half hour to evaluate and interpret the results of colleagues. The model is built and installed fully automatically using CRISP-ML. The development and implementation time of the new ML model is about 2% compared to the data understanding, the development, and implementation of the ML model, and the working time reduced to one 50th alone guarantees a return.

## 5. Conclusions and Future Work

With the development of Industry 4.0 and the opportunities offered by digitalization, it is crucial to bring science and research closer and closer to production, and sensors play an essential role in this. Presently, software sensors are gaining more and more space, which can predict critical parameters that are difficult to measure in production processes. However, software sensors require the development of special ML algorithms that must be continuously monitored, operated, and maintained. The methodology outlined in the scientific paper and the case study discussed in detail present a possible solution for the possibility of using software sensors. The introduction of ML models into production involves several nested components and processes. CRISP-ML is a systematic process model for ML software development that raises the awareness of potential risks and emphasizes quality assurance to reduce these risks to ensure the success of the ML project. The CRISP-ML methodology consists of five parts of a sizeable cyclical process that helps build traditional research and development digitization PoC projects into a thriving, sustainable and long-term system. The main elements of the application of the CRISP-ML methodology are model development, continuous data cleaning, feature engineering, model validation, performance monitoring, and data visualization. The other essential elements of this methodology are edge and cloud computing, which are needed for the continuous development of models, serial data transfer, and onsite access to the models. The ML models used in the two laboratory measurements presented in the case study are suitable for the use of software sensors. Furthermore, the architecture presented is related to the methodology using elements of edge and cloud computing. The ML models presented in this article meet industry requirements and are suitable for estimating parameters. Our next goal is to build similar models to predict as many parameters as possible, which can help ensure quality assurance and better production.

Our future goal is to install software sensors for various process units using the framework to improve manufacturing processes further. The CRISP-ML methodology helps develop models consistently and systematically, and it is essential not to have to develop a separate model for each sensor. In the case of application and monitoring of the developed models, sensor replacements and maintenance can cause problems in the accuracy of the models, and the developed methodology must provide a solution for these (e.g., method and model transfer).

## Figures and Tables

**Table 1:** Results of the 10-fold cross-validation (10-cv) and results of the performance dataset (perf.) nitrogen content of ML models.

Nitrogen Content	*RMSE*		$R^{2}$		*RPD*	
	10-cv	perf.	10-cv	perf.	10-cv	perf.
**PLSR**	0.010	0.035	0.999	0.975	57.73	6.36
**RFR**	0.089	0.084	0.972	0.929	5.98	3.77
**XGBoost**	0.005	0.112	0.999	0.747	31.62	1.98

**Table 2:** Results of the 10-fold cross-validation (10-cv) and results of the performance dataset (perf.) for the quartz content of ML models.

Quartz Content	*RMSE*		$R^{2}$		*RPD*	
	10-cv	perf.	10-cv	perf.	10-cv	perf.
**PLSR**	2.032	2.407	0.900	0.731	3.165	1.930
**RFR**	1.434	4.671	0.621	0.937	1.625	4.010
**XGBoost**	1.966	4.660	0.913	0.870	3.406	2.779

## Data Availability

Restrictions apply to the availability of these data. Data was obtained from MOL Group Plc, and are available from the authors with the permission of MOL Group Plc.

## Abbreviations

The following abbreviations are used in this manuscript:

AI	artificial intelligence
AMI	Amazon Machine Image
CI/CD	continuous integration/continuous delivery
CRISP-DM	CRoss Industry Standard Process for Data Mining
CRISP-ML	CRoss Industry Standard Process for Machine Learning
DevOps	compound of development and operations
DMG	Data Mining Group
DoE	design of the experiment
EDA	exploratory data analysis
ERP	enterprise resource planning
FT-IR	Fourier-transform infrared spectroscopy
HSE	health and safety executive
IoT	internet of things
KPIs	key performance indicators
LIMS	laboratory information management system
LSS	lean six sigma
ML	machine learning
MLOps	machine-learning model operationalization management
NIPALS	nonlinear iterative partial least squares
OEE	overall equipment effectiveness
PAT	process analytical technology
PC	personal computer
PLSR	partial least squares regression
PMML	predictive model markup language
PoC	proof of concept
PRISMA	preferred reporting items for systematic reviews and meta-analyses
PSE	process system engineering
R${}^{2}$	correlation coefficient
RFR	random forest regression
RMSE	root-mean-square deviation
RPD	relative percent differences
SPC	statistical process control
XGBoost	extreme gradient boosting
10-cv	ten-fold cross-validation
